# Relative Nuclease Resistance of a DNA Aptamer Covalently Conjugated to a Target Protein

**DOI:** 10.3390/ijms23147778

**Published:** 2022-07-14

**Authors:** Yudai Tabuchi, Jay Yang, Masumi Taki

**Affiliations:** 1 Department of Engineering Science, Graduate School of Informatics and Engineering, University of Electro-Communications (UEC), Chofu 182-8585, Japan; t1833099@gmail.com; 2 School of Medicine and Public Health, University of Wisconsin, Madison, WL 53706, USA; 3 Department of GI Surgery II, Graduate School of Medicine, Hokkaido University, Sapporo 068-8638, Japan; 4 Institute for Advanced Science, University of Electro-Communications (UEC), Chofu 182-8585, Japan

**Keywords:** covalent aptamer, nuclease resistance, sulfur (VI) fluoride exchange reaction (SuFEx), targeted covalent inhibitor (TCI), middle-molecule covalent drug, covalent biologics, complementary-strand (CS) antidote, reversing adverse drug effects (ADEs), aryl-sulfonyl fluoride warhead

## Abstract

A major obstacle to the therapeutic application of an aptamer is its susceptibility to nuclease digestion. Here, we confirmed the acquisition of relative nuclease resistance of a DNA-type thrombin binding aptamer with a warhead (TBA_3_) by covalent binding to a target protein in the presence of serum/various nucleases. When the thrombin-inhibitory activity of TBA_3_ on thrombin was reversed by the addition of the complementary strand, the aptamer was instantly degraded by the nucleases, showing that the properly folded/bound aptamer conferred the resistance. Covalently binding aptamers possessing both a prolonged drug effect and relative nuclease resistance would be beneficial for in vivo translational applications.

## 1. Introduction

Modifications of the molecular structures of nucleic-acid aptamers [1,2,3,4,5,6,7,8] by incorporation of a non-natural phosphorothioate backbone and manipulation of the sugar or 5′-end structure are often used to reduce rapid hydrolysis by nucleases and to increase the in vivo circulation half-life [1,8,9]. It has also been suggested that unmodified natural DNA aptamers can be protected from hydrolysis by nucleases when they are tightly bound to target proteins. This nuclease-resistant property of a bound aptamer has been exploited to speed up the SELEX process for identifying an aptamer with the desired target specificity. A recent paper reported that an aptamer covalently bound to thrombin through a proximity-driven reaction conferred resistance to degradation by serum [10]. These observations are consistent with the idea that the nuclease resistance of a DNA aptamer depends on its affinity to the target protein [11,12,13,14,15]. However, whether the nuclease resistance of an aptamer depends on the exact chemistry of its covalent binding to the target remains unknown.

**Figure 1:**
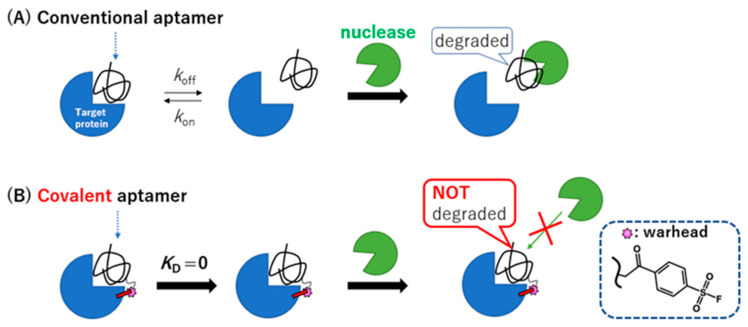
Susceptibility mechanism for the hydrolysis mediated by nucleases. (**A**) Conventional DNA aptamer: the aptamer (folded string) binds to its target protein (blue) (left). The aptamer dissociates from the target protein (middle). The free state of the aptamer is recognized by the nucleases (right). (**B**) Covalently binding DNA aptamer: a warhead-modified aptamer (pink star) forms a covalent bond (left) and permanently binds to the target protein (middle), which causes a semi-permanent nuclease resistance (right).

We recently reported on the creation of a covalently binding DNA aptamer by introducing a SO_2_F warhead (i.e., a reactive group) into designated positions on an aptamer [16]. This method differs from the previously reported inverse nucleophile reaction [10]. The warhead-modified covalently binding aptamer forms a permanent bond to a target protein with a dissociation constant (*K*_D_) rate of effectively zero, resulting in a left-shifted concentration-dependent inhibition activity. Here, we show that the covalently binding DNA aptamer also confers relative nuclease resistance to serum and to other specific nucleases (Figure 1). However, the nuclease sensitivity of the covalently bound aptamer returns with the addition of the complementary-strand antidote DNA, suggesting that the binding of the aptamer to the target confers the nuclease resistance and not the covalent-conjugation per se (Figure S1).

## 2. Results and Discussion

### 2.1. Kinetics of Thrombin Inhibition by TBA_3_ Are Consistent with a Two-Step Process

**Figure 2:**
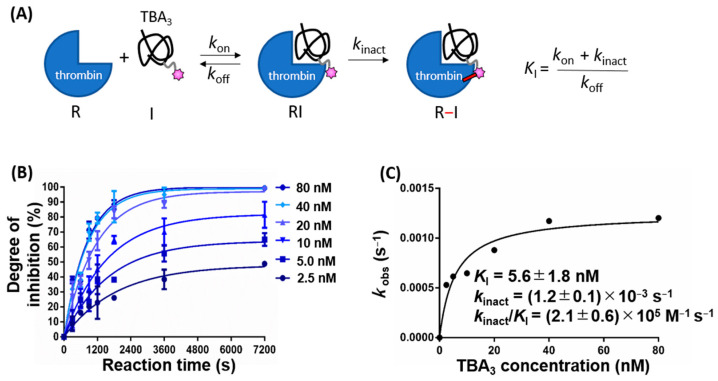
(**A**) Reaction kinetics of TBA_3_: a reversible interaction between TBA_3_ and thrombin is described in terms of rate constants of association (*k*_on_) and dissociation (*k*_off_). Inactivation rate of thrombin by the covalent binding of TBA_3_ is described as *k*_inact_. The resulting *k*_inact_:*K*_I_ ratio is preferred for ranking the potency of covalent inhibitors against a target, rather than using IC50 values [31]. (**B**) Thrombin inhibition at each reaction time was monitored via a turbidimetric assay for different concentrations of TBA_3_. The pseudo-first-order rate constant *k*_obs_ was calculated for each TBA_3_ concentration using nonlinear regression analysis in GraphPad Prism 6. (**C**) The obtained *k*_obs_ values were plotted against each covalent binder concentration to afford the second-order rate constant *k*_inact_/*K*_I_.

As described previously, we introduced the SO_2_F warhead, which can react with any nucleophilic amino acid residues (i.e., serine, threonine, lysine, tyrosine, cysteine, and histidine) [17,18,19,20,21,22,23,24], into the 3rd thymine residue from the 5′-end of the thrombin binding aptamer (TBA), to create the covalently binding TBA (TBA_3_) [16]. We had previously deduced that TBA_3_ is covalently bound to Y88 and H91 residues of thrombin through an LC-MS/MS analysis of trypsin digest fragments [16]. Here, we hypothesize that TBA_3_ reversibly binds to thrombin prior to forming an irreversible covalent bond. This type of two-step process is observed for many known conventional covalent drugs [25,26,27,28,29,30,31] (Figure 2A). First, we demonstrated that TBA_3_ showed a time-dependent inhibition activity, which supports the hypothesized two-step process (Figure 2B). If the irreversible covalent binding step preceded the rapid reversible equilibrium binding (i.e., a pseudo-first-order irreversible binding reaction), the *k*_obs_ vs. TBA_3_ concentration plot would be linear (Scheme A, Figure 9.2 in [32]). TBA_3_ showed a calculated *k*_inact_/*K*_I_ value of (2.1 ± 0.6) × 10^5^ M^−1^s^−1^, which was of the same order as clinically approved covalent drugs (Figure 2C) [33].

### 2.2. TBA_3_ Covalently Bound to Thrombin Resists Degradation by Human Serum

**Figure 3:**
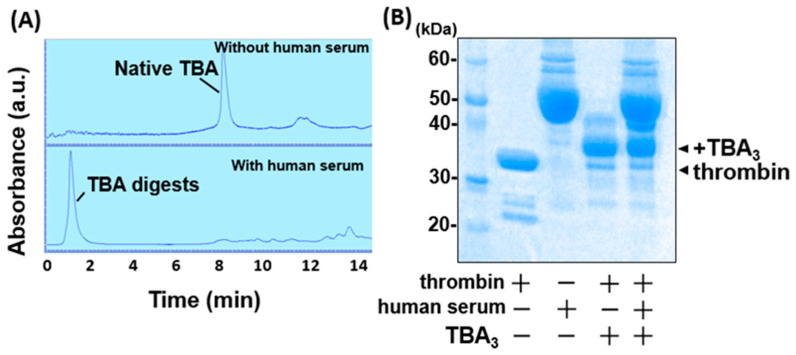
Nuclease resistance of unmodified TBA and TBA_3_ in the presence of human serum. (**A**) LC absorbance profiles of unmodified TBA incubated with thrombin in the absence/presence of human serum. (**B**) Nuclease resistance of TBA_3_ in the presence of human serum, confirmed by 12% (*w*/*v*) SDS-PAGE. Whole proteins were visualized by CBB staining. The band density of TBA_3_-conjugated thrombin (abbreviated as + TBA_3_) did not change after serum incubation for 24 h at 37 °C.

Next, we evaluated the nuclease resistance of TBA_3_ in the presence of human serum or DNases. As a preliminary experiment, the hydrolysis of unmodified TBA was monitored by HPLC. After the serum treatment of TBA in the presence of thrombin, the original TBA peak completely disappeared, and a low-molecular-weight nucleotide peak was detected at the void volume (Figure 3A). As expected, unmodified TBA with a finite off rate dissociated from the target thrombin and was digested by the nucleases in the human serum. Since monitoring the hydrolysis of TBA_3_ covalently conjugated to thrombin by HPLC is problematic, we followed a mobility shift of thrombin due to the covalent binding with TBA_3_ by SDS-PAGE. Thrombin incubated with TBA_3_ resulted in a persistent mobility shift on the gel electrophoresis, where the protein bands were visualized by CBB staining. The shifted band persisted even after incubation with the human serum for 24 h (Figure 3B), which suggests that TBA_3_ covalently bound to thrombin resisted nuclease digestion.

### 2.3. TBA_3_ Covalently Bound to Thrombin Resists Degradation by Both Exonucleases and Endonucleases

**Figure 4:**
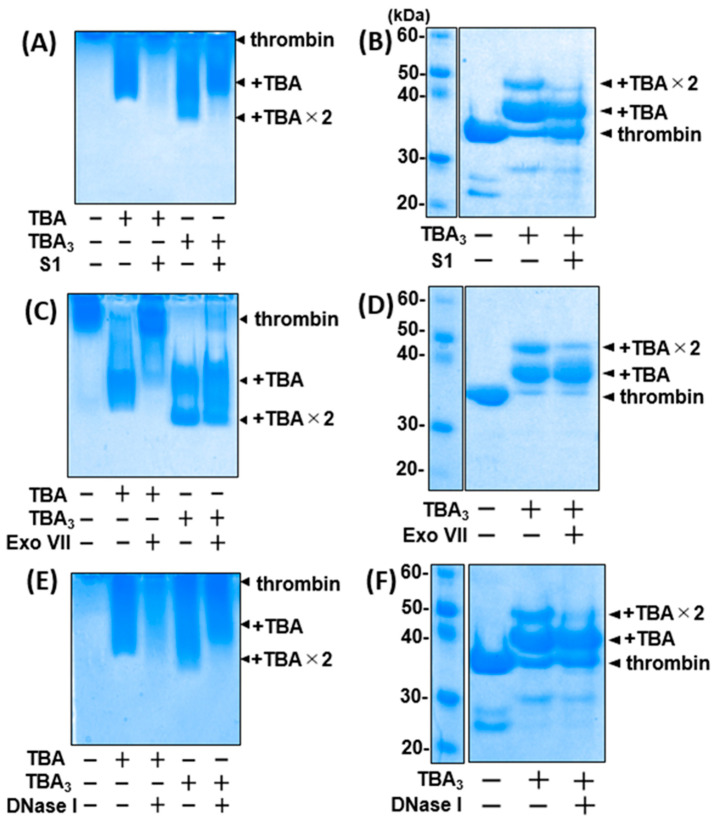
Nuclease resistances of unmodified TBA and TBA_3_ against S1 (**A**,**B**), Exo VII (**C**,**D**), or DNase I (**E**,**F**) nucleases were confirmed by 12% native PAGE (left) and SDS-PAGE (right), respectively. Each nuclease treatment was performed for 24 h at 37 °C, and whole proteins were visualized using CBB staining. TBA monoadduct and bisadduct on thrombin are abbreviated as +TBA and +TBA × 2, respectively.

To assess the nuclease resistance mechanism, we used several purified exonucleases and endonucleases instead of the human serum in further experiments. The nuclease resistance of unmodified TBA or TBA_3_ was evaluated by native PAGE and SDS-PAGE, which reflect the bound and the covalently conjugated states of the aptamer/thrombin (e.g., ‘RI’ and ‘R-I’ states in Figure 2A), respectively. The bands corresponding to TBA-thrombin disappeared when treated with all nucleases, whereas those of TBA_3_-thrombin did not (Figure 4). TBA_3_ induced two distinct mobility shifts corresponding to the TBA_3_ monoadduct and bisadduct via the covalent conjugation. For the bisadduct, two equimolar TBA_3_ moieties were probably conjugated to different residues of thrombin, while both TBA_3_ moieties seemed to recognize the same TBA-binding site [16]. In the presence of nucleases, the monoadduct band remained dense, whereas that of the bisadduct was largely eliminated. This suggests that while one of the conjugated TBA_3_ moieties was in the bound state on thrombin, the other one was microscopically in the unbound state and was recognized by the nucleases (Figure S1, right). The conjugated bisadduct was equally digested with DNase I and S1 endonucleases, or with Exo VII exonuclease (Figure 4), suggesting that TBA_3_ even microscopically displaced from the thrombin binding pocket was no longer afforded steric hindrance and was recognized by the nucleases, similarly to unmodified TBA.

### 2.4. The Complementary-Strand Antidote Restores the Nuclease Sensitivity

**Figure 5:**
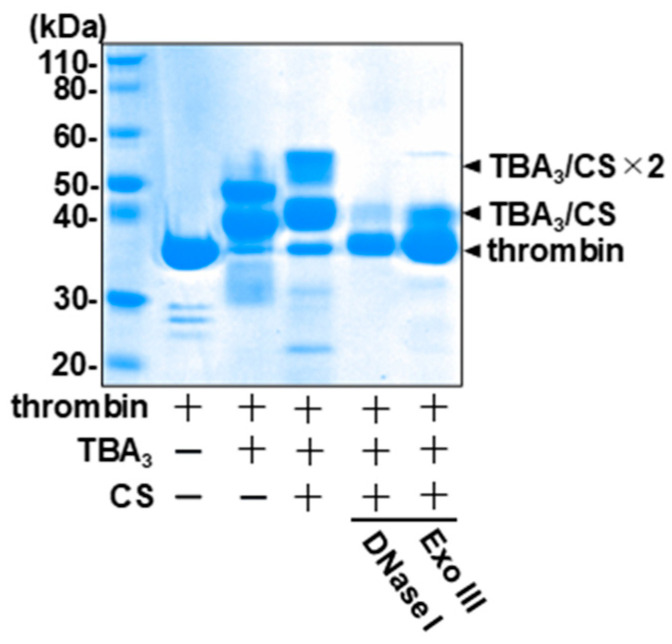
DNase I or Exo III nuclease susceptibility of double-stranded TBA3 with the complementary strand (CS) was confirmed by 12% SDS-PAGE. Each nuclease treatment was performed for 24 h at 37 °C, and whole proteins were visualized using CBB staining.

A unique and highly desirable property of the aptamer drug is its inherent reversibility via the addition of the CS antidote. We have previously shown that the inhibition of the thrombin enzymatic activity by TBA_3_ is reversed by the CS [16], and here we investigated the effect of the CS on the nuclease resistance of TBA_3_. The addition of the CS resulted in a further mobility shift of the TBA_3_-conjugated thrombin band in SDS-PAGE (Figure 5, middle), indicating that the CS formed a double strand with the thrombin-conjugated TBA_3_. The density of the shifted bands was reduced when treated with DNA duplex-specific nucleases (i.e., Exonuclease III and DNase I) (Figure 5, right). This indicated that the CS-hybridized double-stranded TBA_3_ became unbound and was hydrolyzed by the nucleases even while covalently tethered to thrombin (Figure S1, bottom). Direct DNA staining of the gel confirmed the presence of the aptamer in the mobility-shifted bands and a loss of staining upon treatment with DNase I (Figure S2).

### 2.5. Sustained Inhibition of Thrombin Activity by TBA_3_ in the Presence of Nucleases

**Figure 6:**
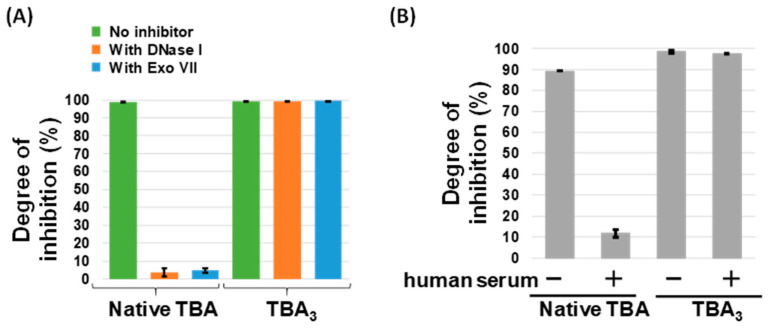
Comparison of thrombin inhibition activity between unmodified TBA and TBA_3_ via the turbidimetric assay. (**A**) Thrombin inhibition activity of each aptamer in the presence of DNase I or Exo VII nuclease. The maximum absorbance (288 nm) of fibrin polymerization at 0 s was normalized to 0%. (**B**) Thrombin inhibition activity of each aptamer in the presence of human serum. Inhibition was calculated as above in (**A**).

Finally, thrombin inhibition by unmodified TBA and TBA_3_ in the presence of nucleases or human serum was monitored by a time-dependent change in optical density at 288 nm (i.e., a turbidimetric assay), which corresponds to thrombin-induced fibrin aggregation [16]. The thrombin inhibition activity of unmodified TBA was decreased when incubated with the nucleases or human serum, whereas that of TBA_3_ was not (Figure 6). This indicates that TBA_3_ resisted nuclease digestion and maintained the target inhibition activity when covalently bound to the target protein.

## 3. Materials and Methods

### 3.1. Chemicals and Reagents

All the reagents and solvents were purchased commercially and used without further purification, including human α-thrombin (Haematologic Technologies Inc., #HCT-0020, Essex Junction, VT, USA), fibrinogen from human plasma (Aldrich, #9001-32-5, St. Louis, MO, USA), human serum (Sigma, #H4522, St. Louis, MO, USA), Exonuclease III (NEB, #M0206S, Ipswitch, MA, USA), Exonuclease VII (NEB, #M0379S, USA), S1 nuclease (Takara, #2410A, Shiga, Japan), and DNase I (Nippon Gene, #314-08071, Tokyo, Japan). Thrombin binding aptamer (TBA): 5′-GGTTGGTGTGGTTGG-3′, alkyne-containing TBA (T_3_): 5′-GGXTGGTGTGGTTGG-3′ (X indicates a 5-Octadinyl-dU possessing a long spacer and a terminal alkyne replacing the thymine residue), and the complementary strand (CS) of TBA: 5′-CCAACCACACCAACC-3′ were synthesized by Integrated DNA Technologies (IDT) Inc. (Coralville, IA, USA).

### 3.2. High-Pressure Liquid Chromatography (HPLC) Analysis

A small-scale quantitative analysis of aptamers was carried out using a reverse-phase semi-micro HPLC system (PU-2085 with a C18 TSKgel column, JASCO with Tosoh #21813, Tokyo, Japan) connected to a photodiode array (PDA) detector. The aptamers were separated using a 0–60% gradient of acetonitrile containing a 20 mM triethylamine acetate aqueous solution (pH 7.4) for 26 min at a flow rate of 200 μL per minute.

### 3.3. Image Capturing

All images of stained gel and in-gel fluorescence were captured by ChemDoc XRS+ (Bio-Rad Laboratories Inc., Hercules, CA, USA), and band intensities were quantified using Image Lab 3.0.1 software (Bio-Rad Laboratories, Inc.).

### 3.4. Synthesis of a Covalent Aptamer: TBA_3_

TBA_3_ with the SO_2_F warhead introduced at the 3rd T residue was synthesized according to the following procedure [16]. Tris(3-hydroxypropyltriazolylmethyl)amine (in water, 0.50 μmol, Aldrich, #762342, USA) and copper (II) sulfate (in water, 0.25 μmol, Aldrich, #451657, USA) were mixed at a 5 μL scale and incubated at room temperature for 5 min. Then, alkyne-containing TBA (T_3_) (in water, 10 nmol), 4-(2-azidoacetyl)-benzene-1-sulfonyl fluoride (in DMSO, 0.50 μmol), and ascorbic acid (in water, 0.40 μmol, Aldrich, #A92902, USA) were successively added, and the mixture (25 μL in total) was reacted for 1 h at 4 °C. The crude reaction product was purified by ethanol precipitation as follows. Sodium acetate (in water, 9 μmol) and cold ethanol were added to the crude product and incubated at −20 °C for 1 h. After centrifugation (12,600× *g*, 20 min, 4 °C), the supernatant was removed, and the pellet was washed with 70% ethanol. The residue was dissolved in nuclease-free water.

### 3.5. Kinetics Evaluation of TBA_3_

Thrombin activity inhibition by TBA_3_ was measured using a turbidimetric assay. We mixed various molar concentrations of TBA_3_ with a constant molar concentration of thrombin (25 μM), with various incubation times in Dulbecco’s phosphate-buffered saline (D-PBS) at 37 °C. Then, each reaction mixture was added into fibrinogen solution (in D-PBS) to give a final concentration of 2.5 nM thrombin and 1 mg/mL of fibrinogen, and the maximum absorbance of the polymerized fibrin (288 nm) was measured after three minutes using a nanophotometer (Implen, Munich, Germany) with a 10 mm plastic cell. In each experiment, the maximum absorbance of polymerized fibrin at 0 s was normalized to 0, and the relative absorbance was quantified. The thrombin inhibition denoted as degree of inhibition (DoI), was calculated as the mean ± SD (n = 3) from the quantified values and plotted against time. Exponential curve fitting of the plot using GraphPad Prism 6 (GraphPad Software, San Diego, CA, USA) gave us the value of the observed rate constant (*k*_obs_) at each TBA_3_ concentration via the equation DoI (%) = *v*_i_/*k*_obs_ × [1 − exp (−*k*_obs_ × t)], where *v*_i_ = initial velocity (Figure 2B). Finally, the values of *k*_obs_ (i.e., the pseudo-first-order rate constant) were plotted against the TBA_3_ concentrations, and the values of *K*_i_ and *k*_inact_ (i.e., the inhibition constant and inactivation rate constant, respectively) were determined by curve fitting of *k*_obs_ = (*k*_inact_ × [*I*])/(*K*_i_ + [*I*]); [*I*] = TBA_3_ concentration (Figure 2C).

### 3.6. Evaluation of Nuclease Resistance by Liquid Chromatography or Gel Electrophoresis

Unmodified TBA or TBA_3_ (0.10 mM) was mixed with thrombin (25 μM) in D-PBS and incubated for 3 h at 37 °C. Then, human serum was added to 40% (*v*/*v*) and further incubated for 24 h at 37 °C. The reaction mixture was heated at 95 °C for 10 min and centrifuged at 10,000× *g* for 10 min. The supernatant was analyzed by HPLC (Figure 3A). For the gel electrophoresis analysis, Sample Buffer (Wako, #198-13282, Osaka, Japan) supplemented with 2-mercaptoethanol was added to the reaction mixture, and the mixture was separated by 12% sodium dodecyl sulfate-polyacrylamide gel electrophoresis (SDS-PAGE). Whole proteins were visualized using Coomassie brilliant blue (CBB) staining (Figure 3B). For the assessment of specific nuclease resistance, ten units of specific nuclease (S1 nuclease, Exonuclease VII, or DNase I) were added instead of the serum (Figure 4).

### 3.7. Evaluation of Nuclease Resistance of TBA_3_ after the Addition of the Complementary Strand (CS) by Gel Electrophoresis

Thrombin (25 μM), with or without TBA_3_ (0.10 mM) in D-PBS, was incubated for 3 h at 37 °C. The mixture was supplemented with or without CS (400 μM) for 30 min at 37 °C. Then, it was mixed with ten units of each nuclease (DNase I, S1 nuclease, Exonuclease VII) for 24 h at 37 °C. Then, as described in the previous procedure (Section 3.6), SDS-PAGE was performed, and whole proteins were visualized by CBB staining (Figure 5).

### 3.8. Evaluation of Thrombin Inhibition Activity in the Presence of Human Serum or Nucleases

For the assessment of thrombin inhibition activity, TBA or TBA_3_ (0.10 mM) was mixed with thrombin (25 μM) in D-PBS and incubated for 3 h at 37 °C. Then, ten units of either DNase I or Exonuclease VII, or 40% (*v*/*v*) human serum was added. The mixture was further incubated for 24 h at 37 °C. The thrombin activity of each reaction mixture was analyzed using a turbidimetric assay. Each reaction mixture was added into the fibrinogen solution (in D-PBS) to give a final concentration of 2.5 nM thrombin and 1 mg/mL of fibrinogen. The maximum absorbance of polymerized fibrin (288 nm) was measured after three minutes using the nanophotometer. In each experiment, the maximum absorbance of polymerized fibrin at 0 s was normalized to 0, and the relative absorbance was quantified (Figure 6).

## 4. Conclusions

In conclusion, we demonstrated a novel method of endowing a DNA aptamer with nuclease resistance. TBA_3_ formed a permanent bond to thrombin, resulting in a drug–protein complex that was not affected by the classical equilibrium kinetics of binding (i.e., *K*_D_ = 0). As a result, TBA_3_ showed a long-term relative resistance against nucleases and maintained the desired thrombin inhibition activity. While we did not examine the in vivo pharmacokinetics of TBA_3_, a prolonged inhibition of the target protein is expected from the extension of the pharmacological half-life due to the covalent binding, regardless of the macroscopically observable pharmacokinetic half-life of the free TBA_3_. For in vivo applications, the relatively slow covalent bond formation by TBA_3_ could be easily overcome by a continuous infusion, to maintain an adequate serum concentration analogous to other short-acting drugs. We believe these advantages provided by the covalently binding aptamer will mitigate the major obstacles to the therapeutic application of aptamers, such as susceptibility to hydrolysis by nucleases and rapid clearance through glomerular filtration, while maintaining the highly desirable property of a reversible covalent drug, and will speed up the translation of aptamer therapeutics to clinical applications.

## Data Availability

The data presented in this study are available on request from the corresponding author. The data are not publicly available due to privacy restrictions.

## Supplementary Materials

The following supporting information can be downloaded at: https://www.mdpi.com/article/10.3390/ijms23147778/s1. References [34,35] are cited in the supplementary materials.
