# Supplementary material for: Relative Nuclease Resistance of a DNA Aptamer Covalently Conjugated to a Target Protein
Source: Int J Mol Sci. 2022 Jul 14;23(14):7778. doi: 10.3390/ijms23147778 (PMC9319527; doi:10.3390/ijms23147778)
Supplement: Supplementary file 1 [file ijms-23-07778-s001.zip › ijms-1785233-supplementary.pdf]

Supplementary Figures

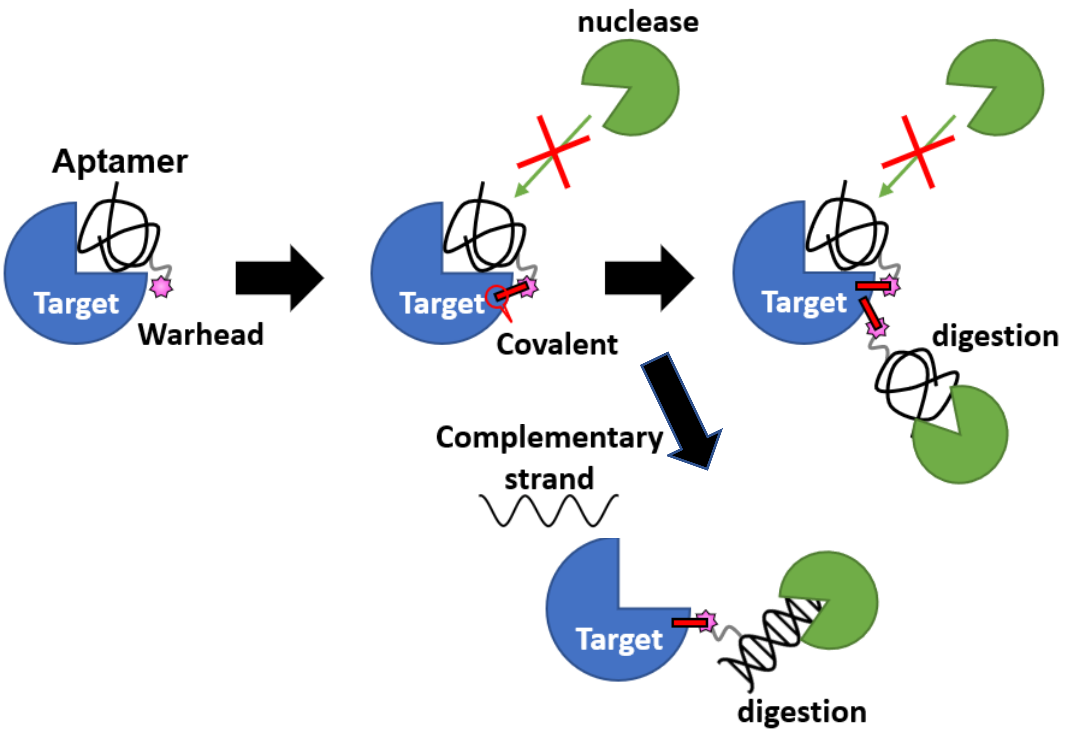

Figure S1 *Summary of this work: a cartoon depicting a potential nuclease resistance mechanism of target-bound TBA$_3$.* A warhead-modified aptamer (left) forms a covalent bond and permanently binds to the target protein resulting in a nuclease resistance state (middle). When two TBA$_3$ molecules are conjugated to the target forming a bis-adduct sharing the same binding site, one of TBA$_3$ in a microscopically unbound-state is recognized by the nuclease and digested (right). In the presence of the complimentary strand antidote (bottom), the aptamer still covalently conjugated to the target is displaced from the binding site. The unbound double-stranded TBA$_3$ is recognized by the nuclease and digested.

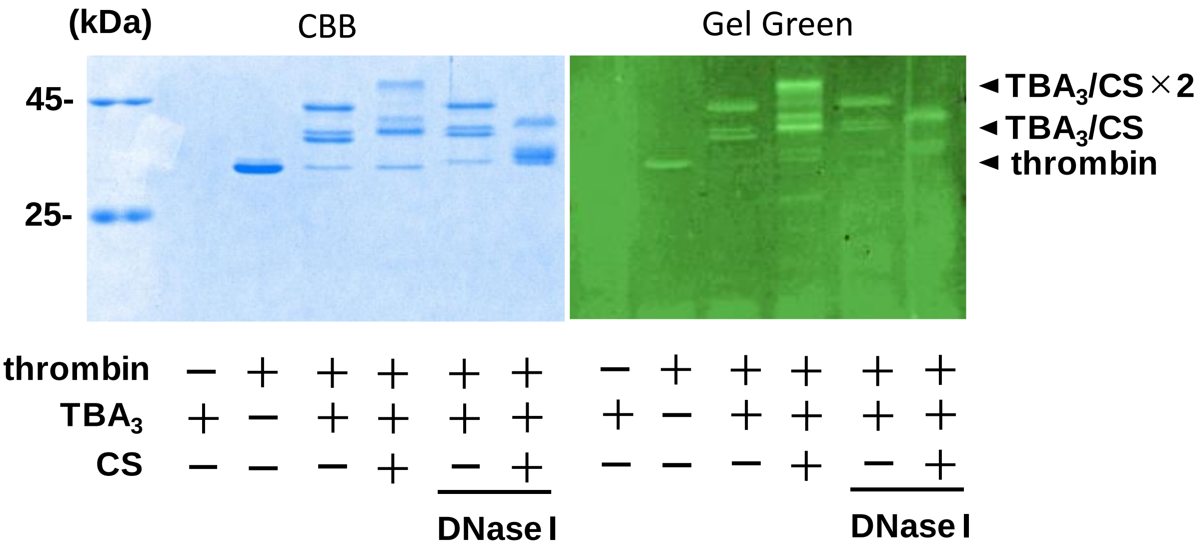

Figure S2 *DNA and Protein stain of a denaturing SDS gel.* Thrombin with or without TBA_3 and CS were subjected to DNase I digestion, as in Figure 5. DNA was visualized by a GelGreen stain (EMD Millipore, SCT125) (right) followed by a Coomassie brilliant blue protein stain (left) of the same gel. Arrows indicate the unbound thrombin, thrombin bound with TBA_3 and CS (TBA_3/CS), and thrombin bound to two TBA_3 and CS (TBA_3/CS x 2). When a double-stranded TBA_3 was formed by the addition of CS, the fluorescence intensity of GelGreen became the brightest, most probably due to its intercalation [1] to the double-helix. The brightest bands of the intercalated GelGreen disappeared when DNase I digested the double-stranded TBA_3. Such a DNA intercalator very weakly stains a protein (i.e., thrombin) as a background signal [2].

1. Crisafuli FAP, Ramos EB, Rocha MS. Characterizing the interaction between DNA and GelRed fluorescent stain. *European Biophysics Journal with Biophysics Letters*. 2015;44(1-2):1-7.
2. Hirasawa S, Kitahara Y, Okamatsu Y, Fujii T, Nakayama A, Ueno S, et al. Facile and Efficient Chemoenzymatic Semisynthesis of Fc-Fusion Compounds for Half-Life Extension of Pharmaceutical Components. *Bioconjugate Chemistry*. 2019;30(9):2323-31.
